# Accelerated Editing Method for Vehicle Durability Fatigue Load Spectrum Based on Wigner-Ville Transform

**DOI:** 10.3390/s23146435

**Published:** 2023-07-16

**Authors:** Yongle Yang, Zhifei Zhang, Liangfeng Peng, Jie Jin, Qinghua Wang

**Affiliations:** 1 College of Mechanical and Vehicle Engineering, Chongqing University, Chongqing 400030, China; yl.yang@cqu.edu.cn (Y.Y.);; 2 Research Institute of Highway Ministry of Transport, Beijing 100088, China; 3 Xiangyang Daan Automobile Test Center Co., Ltd., Xiangyang 441004, China

**Keywords:** load spectrum editing, Wigner-Ville transform, instantaneous energy spectrum, genetic algorithm, fatigue simulation

## Abstract

A Wigner-Ville transform-based (WVT-based) load spectrum fast editing method for vehicle parts is proposed to improve the efficiency of durability tests. In this method, the instantaneous energy spectrum (IES) of the original time-domain signal is obtained via the Wigner-Ville transform, which is used as a criterion to identify time-domain points of ineffective damage contribution. A genetic algorithm (GA) based threshold optimization model is also proposed to automatically set the threshold of the IES under consideration of the relative damage requirements and statistical parameters of the signal. The effectiveness of the above proposed editing method is demonstrated by compiling an SUV’s suspension coil spring signal obtained from physical sensor-based measurements. Meanwhile, the same spectrum is also processed using time-domain editing, Short-time Fourier-transform, and S-transform methods for comparison. The results show that the WVT-based edited spectrum has a time-duration retention ratio of about 76.30%, which is significantly superior to other methods, with the same pseudo-damage retention and statistical parameter error constraints. Moreover, in combination with the fatigue simulation analysis, it verifies that the load effect of the edited spectrum matches well with that of the original. Thus, the proposed method is considered more effective for compiling component load signals in vehicle acceleration durability tests.

## 1. Introduction

Durability analysis of vehicle components represents a crucial aspect of the product development process conducted by vehicle manufacturers. The load spectrum applied to the components is critical for the analysis of fatigue life tests [1]. For the measured load signals, a rational and efficient preparation method for accelerated editing can effectively improve the efficiency of the fatigue test analysis. Currently, accelerated editing methods include two main categories: time-domain and frequency-domain editing methods.

Traditional time-domain editing methods encompass peak-to-valley extraction, increasing the input vibration amplitude or the applied forcing frequency, and eliminating irrelevant load cycles of test signals [2]. To establish a threshold for load magnitude, Stephens et al. [3] creatively defined the Smith–Watson–Topper parameter as a threshold criterion, then edited the spectrum by deleting time windows below the determined threshold that contained minimal damage. Subsequently, the principle of ‘time damage retention’ [4] has been exploited to achieve signal compression by removing periods with low damage. Kadhim et al. [5] developed a fatigue data editing method that relied on damage values obtained from nCode durability analysis software, and then successfully compressed a load spectrum obtained from the lower suspension arm. Yu et al. [6] proposed a concatenation of the multi-section minimum standard deviation (CMSD) spectrum, followed by a detailed analysis of the CMSD spectrum using the correlated fatigue damage analysis method. Zhou et al. [7] introduced an accelerated compilation method of multiaxial rain-flow projection filtering based on the damage equivalence principle for multiaxial load spectrums. The experimental results demonstrated that it could significantly reduce the time duration while better preserving the frequency-domain characteristics of the original load.

However, there are some drawbacks to the time-domain editing method due to the influence caused by the length of time window. The deleted time segments, for example, can significantly alter the frequency component characteristics of the signal, such as amplitude peaks and frequency content, which are significant in determining accurate fatigue signals [8]. More effective time-frequency processing methods have been investigated to avoid these shortcomings. The damage contribution distribution of the load signal is typically proportional to factors such as amplitude, energy, and so forth [9]. The signal transform analysis method can effectively combine the time-domain data with the frequency-domain characteristics. The Short-time Fourier-transform (STFT) based approach divides the load spectrum into a series of equal-length short segments, and then Fourier-transforms of each segment are adopted to express the load history in the time-frequency domain [10]. Pratumnopharat et al. [11], Liu et al. [12], and Wen et al. [13] successively investigated various fatigue data editing methods, where the accumulative power spectral density (AccPSD) of the signal obtained by STFT was proposed to identify fatigue damage events. The results indicated that the STFT-based method could not only improve the accuracy of fatigue damage retention but also shorten time durations. However, a fixed window length is commonly used in the STFT-based editing method, which leads to some unavoidable limitations.

Furthermore, the wavelet transform (WT) is considered more suitable for detecting amplitude variations due to its variable window length. Based on the discrete-wavelet transform (DWT) theory, Oh et al. [14], Abdullah et al. [15,16], and Zheng et al. [17] used different orders of Daubechies wavelets to identify and extract large damage segments from load spectrums, respectively. Mohseni et al. [18] proposed a fast wavelet transform-based editing method that combined an appropriate optimization strategy alongside with a robust editing process based on DWT, which could solve the issue of the inconsistent lengths of the edited signals. Zheng et al. [1,9], Putra et al. [19,20,21], and Liu et al. [22], on the other hand, acquired the AccPSD via continuous-wavelet transform (CWT) based on the Morlet wavelet. CWT differs from DWT mainly in the discretization of the scale parameter, which is defined as the sum over all times of a signal multiplied by the scale. As such, both different forms of wavelet functions and different layers of wavelet decomposition can make a great difference to the editing effect, which must be determined through trial and error. The S-transform (ST) is an invertible time-frequency spectral localization technique that combines elements of both the WT and STFT. It is widely applied for random signal analysis due to the advantage that it provides multi-resolution analysis while preserving the absolute phase information of each frequency. Abdullah et al. [23] and Dong et al. [24] examined the utilization of the ST to extract the S-T spectrum or maximum amplitude spectrum from load spectrum for detecting damage events contained in fatigue signal. Deficiently, the S-transform’s large computational size comes at a cost, which makes balancing analytical correctness and computational economy problematic.

In general, the loads to which the vehicle is subjected during driving are typically nonstationary random signals. As previously stated, when employed for accelerated editing of load spectrum, the STFT, WT, and ST methods often lack a definitive selection of parameters, necessitating frequent debugging and optimization, resulting in the increase in processing time costs. In contrast, the Wigner-Ville (WV) transform distribution is a bilinear time-frequency distribution that offers an effective analysis of non-stationary random signals without involving the selection of parameters such as window functions, and thus does not suffer from the limitations of the above defects. The WV distribution analysis is widely used in structural non-stationary signal analysis, fault condition monitoring, damage detection, etc. [25,26,27,28] Levy et al. [25,26] proposed a new WV distribution technique-based time-frequency metric for the characterization of frequency sources, which could be used to solve problems of amplitude fluctuations, phase noise, and frequency instabilities caused by the physical mechanisms of such as the oscillators, clocks, etc. Singru et al. [27] examined an experimental method for condition monitoring of bearing failures by utilizing experimental vibration signatures. It showed that the WV distribution analysis is more accurate and effective in both time and frequency domain numerical characterization of the obtained vibration signals than other methods, such as FFT. Andrzej [28] put forward a novel non-parametric damage identification algorithm based on the WV distribution, which exhibits high precision of localization and quantification in damage detection. It also revealed that the WV distribution analysis possesses the advantage that there is no need for parameter selection in the algorithm such as wavelet type and its order, as well as the possibility to reduce the boundary impact. Consequently, it is deemed more advantageous than wavelet-based approaches. Similarly, this advantage is believed to be applicable in the field of load spectrum editing as well.

In this study, to develop a new effective time-frequency-domain accelerated editing method, a Wigner-Ville Transform-based (WVT-based) analysis technology is proposed for generating an instantaneous energy spectrum (IES) from the signal. The instantaneous energy of each corresponding time-domain point serves as a discriminant for determining the magnitude of damage contribution from different amplitude loads. To optimize the threshold selection model for the IES, a genetic algorithm is employed. The load spectrum of a vehicle suspension coil spring is measured and used as an example to validate the novel editing method’s effectiveness and superiority in terms of damage equivalency, characteristic parameters, frequency-domain distribution, fatigue simulation test, etc.

## 2. Methodology

### 2.1. Wigner-Ville Transform Distribution

The Wigner distribution was first proposed by Wigner [29] in 1932 in the quantum mechanics field, and later extended by Ville (1948) [30] to form WV distribution in the signal analysis and processing field. The WV distribution is defined as a function of the energy transform with time and frequency distribution.

The WV distribution is a special type of quadratic nonlinear, also known as bilinear time-frequency transform. It is characterized by its conciseness and ability to provide a high-resolution instantaneous energy density in both the time and frequency domains. Mathematically, the WV distribution is defined as:

(1) $$W_{x}(t,f)=\int_{-\infty }^{\infty }x(t+\frac{\tau }{2})\;x^{*}(t-\frac{\tau }{2})e^{-j2\pi f\tau }d\tau$$

where x(t) is the continuous signal and $x^{*}\left(t\right)$ is the complex conjugate of x(t). The f denotes the spatial frequency variable, τ is the integration variable, and $x\left(t+\frac{\tau }{2}\right)x^{*}\left(t-\frac{\tau }{2}\right)$ is the signal’s instantaneous correlation function.

The WV distribution usually adopts an analytic signal as an input, so analytical signal z(t) of a real signal x(t) is defined as:

(2) $$\begin{array}{l} z(t)=x(t)+jH[x(t)];\\ H[x(t)]=\frac{1}{\pi }\int_{-\infty }^{\infty }\frac{x(\tau )}{t-\tau }d\tau \; \end{array}$$

where H [x(t)] is the Hilbert transform [31].

The advantage of using the analytic signal is that it effectively eliminates the influence of negative frequency components in real signals, thus avoiding the frequency domain mixing. For the analytic signal z(t), the WV distribution is given by:

(3) $$W_{z}(t,f)=\int_{-\infty }^{\infty }z(t+\frac{\tau }{2})\;z^{*}(t-\frac{\tau }{2})e^{-j2\pi f\tau }d\tau$$

where $W_{z}\left(t,f\right)$ is Wigner-Ville energy density distribution of analytic signal.

This shows that the window function selection is not included in the Wigner-Ville transform process, thus avoiding restriction by the uncertainty principle as in STFT. Additionally, it can avoid the limitations inherent in linear time-frequency analysis, such as that the time and frequency resolution are mutually restrained and cannot be considered simultaneously.

By definition, the WV distribution is real-valued, i.e., the self-WV distribution of any signal is a strictly real-valued function of t and f, as follows:

(4) $$W_{z}(t,f)=W_{z}^{*}(t,f)$$

The integration of WV distribution in the plane of time t and frequency f (time-frequency plane) characterizes the energy of the analyzed signal:

(5) $$\int_{-\infty }^{\infty }\int_{-\infty }^{\infty }W_{z}(t,f)dtdf=E$$

According to Equation (5), it is known that WV distribution includes an energy distribution property. That is, the integral of WV distribution along the time axis is equal to the instantaneous energy at a certain frequency:

(6) $$\int_{-\infty }^{\infty }W_{z}(t,f)dt={\left|Z(f)\right|}^{2}$$

Meanwhile, the WV distribution also possesses a Time-Marginal property, where the integral along the frequency axis is equal to the instantaneous energy of the signal at a given moment in time, such that:

(7) $$\int_{-\infty }^{\infty }W_{z}(t,f)df={\left|Z(t)\right|}^{2}$$

In fact, the solution to Equation (6) is essentially the power spectral density information of the signal, while that to Equation (7) is essentially the instantaneous power information.

Usually, signal processing of measured loads uses the discrete form of physical signal. Therefore, the practical calculation will be in the form of discrete WV distribution. The WV distribution in discrete form is defined as [32]:

(8) $$W_{z}(n,m)=t_{s}\sum_{k=-k_{n}}^{k_{n}}z\left(n+\frac{k}{2}\right)z^{*}\left(n-\frac{k}{2}\right)e^{-2\pi imk/\left(2N\right)}$$

where $W_{z}\left(n,m\right)$ is the discrete WV distribution, $k_{n}=\min\;\left[2n,2N-1-2n\right]$, N denotes the number of samples with sampling frequency $f_{s}$, the time and frequency axes in physical units are $t_{n}=t_{s}\times n\left(t_{s}=1/f_{s}\right)$ and $f_{m}=f_{s}\times m/\left(2N\right)$, and n, m represent index numbers for time and frequency vectors, respectively.

Equation (8) is designed for discrete-time signals, which is extremely similar to the original continuous version. This research is carried out based on this formula combined with the MATLAB toolbox.

### 2.2. Accelerated Editing Process via WVT-Based Method

**Figure 1:**
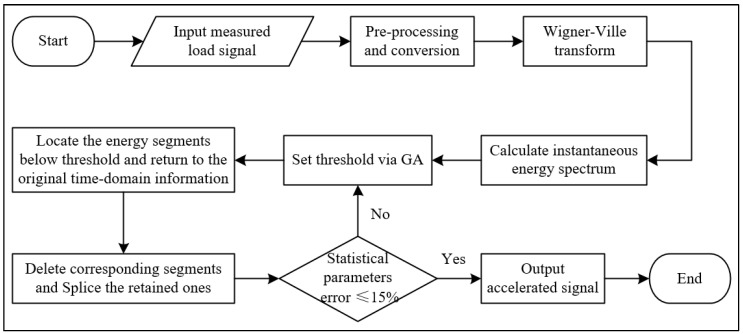
WVT-based accelerated editing process.

In load signal editing, the time duration can be reduced by eliminating small magnitude segments that cause negligible or minimal damage to the component. This paper proposes a concept of applying instantaneous energy to identify fatigue damage events contained in the time history. The larger the instantaneous energy at each time point, the greater the contribution of that load amplitude to vehicle component damage. According to the Time-Marginal property of the WV distribution, the instantaneous energy spectrum can be calculated using the Wigner-Ville transform, which reflects the energy distribution of the load spectrum along the time axis. Figure 1 illustrates the WVT-based accelerated editing process, which consists of the following specific steps:

(1) Inputting of the pre-processed signal x(t) in MATLAB.
(2) Normalization of the signal x(t) to zero mean $x^{\prime }\left(t\right)$. The signal is then transformed into analytic signal z(t) by Equation (2) to eliminate the effect of negative frequency components and make the creation of a real-valued WV distribution matrix easier.
(3) Execution of the Wigner-Ville transform. The Wigner-Ville time-frequency transform is applied to the analytic signal z(t) using the time-frequency toolbox’s ‘tfrwv’ function. After that, a two-dimensional time-frequency real-valued distribution matrix, whose expression is shown in Equation (9), can be generated.

(9) $$W_{z}(n,m)=\left[\begin{array}{cccc} a_{11} & a_{12} & \cdots & a_{1n}\\ a_{21} & a_{22} & \cdots & a_{2n}\\ \vdots & \vdots & & \vdots \\ a_{m2} & a_{m2} & \cdots & a_{mn} \end{array}\right];\;m=1,2,\cdots ,F;\;n=1,2,\cdots ,T$$

where $a_{mn}$ denotes the amplitude at corresponding time $t_{n}$ and frequency $f_{m}$. F and T are the dimensions of obtained two-dimensional matrix that are related to input signal.

(4) Calculation of the instantaneous energy spectrum. Combining the Time-Marginal property of Equation (7), a two-dimensional time-frequency real-valued matrix is integrated along the frequency axis at each time point according to Equation (10) to calculate the corresponding instantaneous energy value of each time point. Then the combined sequence P provides the IES information:

(10) $$\left\{ \begin{array}{l} P=\left[p_{1},p_{2},\cdots p_{n}\right],n=1,2,\cdots ,T;\\ p_{n}=\int_{0}^{f}W_{z}(t_{n},f)df \end{array}\right.$$

where $p_{n}$ is the instantaneous energy value obtained by integrating along the frequency axis at corresponding time point $t_{n}$.

(5) Threshold selection. An optimal threshold is set for the IES to locate the energy points below the threshold that contain no/minor damage contribution.
(6) Accelerated spectrum acquirement. Based on the distribution of the IES, the positions of energy points below the threshold are mapped to the positions of the original time-domain data. Then time-domain points at the same positions (contain no/minor damage contribution) are removed and the remaining ones are spliced to obtain an accelerated spectrum.

### 2.3. Threshold Optimization with GA

Threshold selection determines the efficiency of accelerated editing, which must take into account both the compression efficiency and feature parameters error.

The primary judge index for time duration compression is damage. The pseudo-damage theory is a popular evaluation criterion for damage characteristics, characterizing the potential ability of the load spectrum to carry energy for causing damage to components. Combining the standard S-N curve and Miner’s linear damage accumulation criterion without average stress correction, the pseudo-damage value is calculated as [33]:

(11) $$D=\sum \frac{n_{i}}{N_{i}}$$

where D is total damage, $n_{i}$ is the number of cycles for a certain stress level, and $N_{i}$ is the number of cycles to fracture at that stress level.

Additionally, there are three crucial statistical characteristics for feature representation, namely mean value, RMS, and kurtosis value. The mean value of the load spectrum significantly affects the structural fatigue life. RMS represents the physical average power contained in the random signal, characterizing the average magnitude of energy that is carried by loads. Kurtosis is a fourth-order statistical parameter in the dimensionless amplitude domain that characterizes the peak value of the probability density distribution curve at the mean. To ensure that the loading effects and statistical features do not differ too much after editing, engineering generally requires the pseudo-damage ratio to be above 97%, while the relative errors of statistical parameters should be below 15% [34].

In this paper, an accelerated threshold optimization method based on a GA is proposed. GA [35] is a method to search for the optimal solution by simulating the natural evolution process. Better optimization results can be obtained quickly when solving complex combinatorial optimization problems. The threshold value $p_{0}$ of IES is used as the design variable, and the objective function is the time duration ratio $\min\left(L\left(p_{0}\right)\right)$ before and after editing. To ensure the validity of the accelerated spectrum, two constraints are imposed together: the pseudo-damage retention ratio (Dam) should be greater than or equal to 0.97, and the errors of the three statistical parameters (Δ) should be below 15%. The genetic algorithm is performed to search for an optimistic threshold in the interval of [0,max p(n)] in the IES. Denote the original load as $x_{0}\left(t\right)$ with a time length of $L_{0}$, and denote the accelerated one as $x_{1}\left(t\right)$ with a time length of $L_{1}$. The threshold optimization model can be expressed as:

(12) $$\left\{ \begin{array}{l} \min\\ L(p_{0})=L_{1}/L_{0}\\ s.t.\\ c(1)=0.97-Dam\leq 0\\ c(2)=\Delta avr-0.15\leq 0\\ c(3)=\Delta ku-0.15\leq 0\\ c(4)=\Delta rms-0.15\leq 0 \end{array}\right.$$

where $Dam=\left|\frac{dam\left(x_{1}\left(t\right)\right)}{dam\left(x_{0}\left(t\right)\right)}\right|$ is the pseudo-damage retention ratio, and Δavr, Δku, Δrms are the relative errors of mean, kurtosis and RMS, respectively.

## 3. Load Signals Acquisition and Accelerated Editing

In this section, the durability load signals of an SUV were collected at a test site and one of the suspension coil spring loads was used as an example for accelerated editing to validate the effectiveness of the proposed method.

### 3.1. Load Signals Acquisition and Processing

The SUV test was conducted on a professional proving ground, and the load signals were collected utilizing various professional physical sensors and other equipment. This involves the KISTLER wheel center sextants, strain gauges, acceleration sensors, GPS sensors, and the SoMat eDAQ data acquisition system.

**Figure 2:**
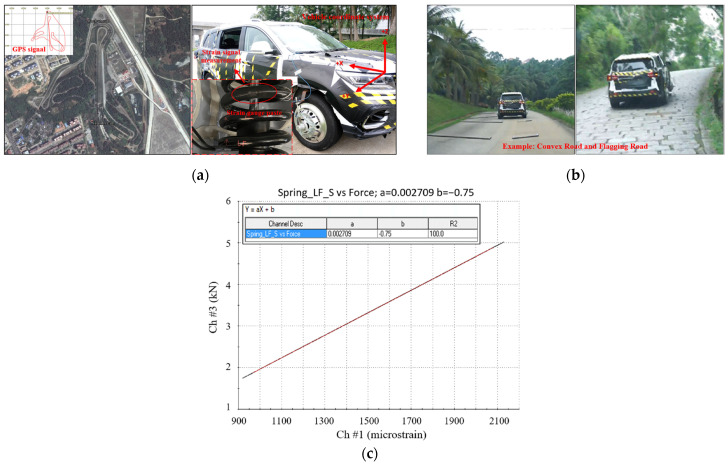
Load signal acquisition: (**a**) Test site roads distribution and Test vehicle; (**b**) Some testing scenarios; (**c**) The front left suspension spring calibration curve (strain-force).

The coil spring signals were tested and collected by employing strain gauges, which were plastered and bridged to establish the relationship between force and bridge circuit output. By fitting the force to the output voltage of the bridge circuit, the calibration coefficient of the spring was obtained. Then, the calibration force under test road conditions could be calculated based on the measured strain data. The test site distribution and test vehicle are shown in Figure 2a, and some of the test scenarios are depicted in Figure 2b. Additionally, the load calibration curve of the left front coil spring signal is shown in Figure 2c.

**Figure 3:**
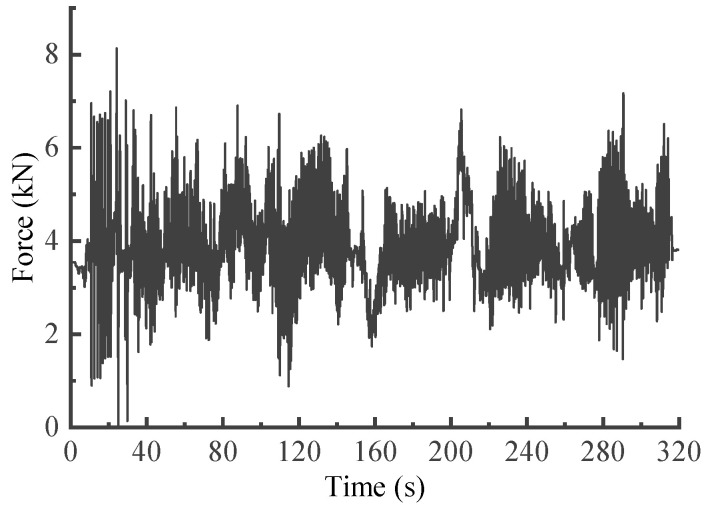
Suspension spring force signal obtained on enhanced test site roads.

After calibration, the suspension coil springs with strain gauges attached were mounted on the vehicle and subsequently tested. The eDAQ data acquisition system was employed with a sample rate $f_{s}$ set at 500 Hz. However, the collected signals often encompass extraneous signal components that hinder analysis [1]. Therefore, referring to the processing flow outlined in the literature [22], the measured load signals were sequentially pre-processed with the corresponding signal preprocessing modules in nCode Glyphworks software (HBM nCode v2018). By selecting suitable methods and parameters, the signals underwent pre-processing steps that encompassed 0–50 Hz low-pass filtering, drift correction, deburring and 200 Hz resampling process, and the reasonableness of the processed signals was then checked. Additionally, conspicuous transition sections were removed. The pre-processed signal is shown in Figure 3, with a total time duration of 320 s.

### 3.2. Accelerated Editing via WVT-Based Method

**Figure 4:**
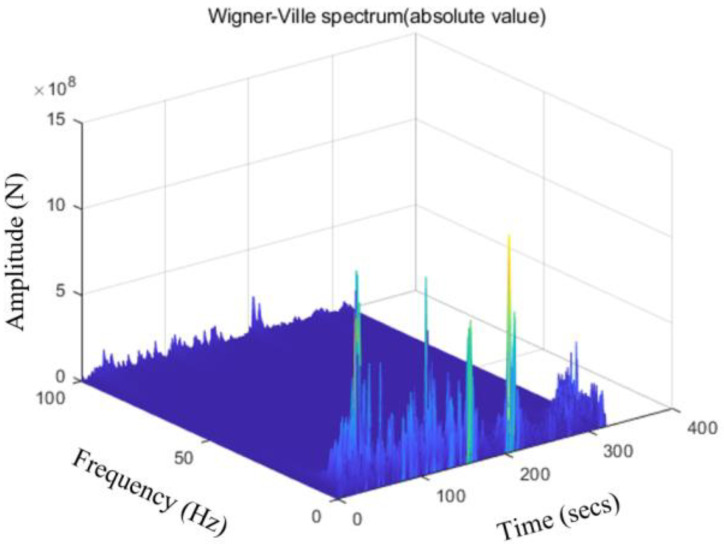
Wigner-Ville spectrum (absolute value).

**Figure 5:**
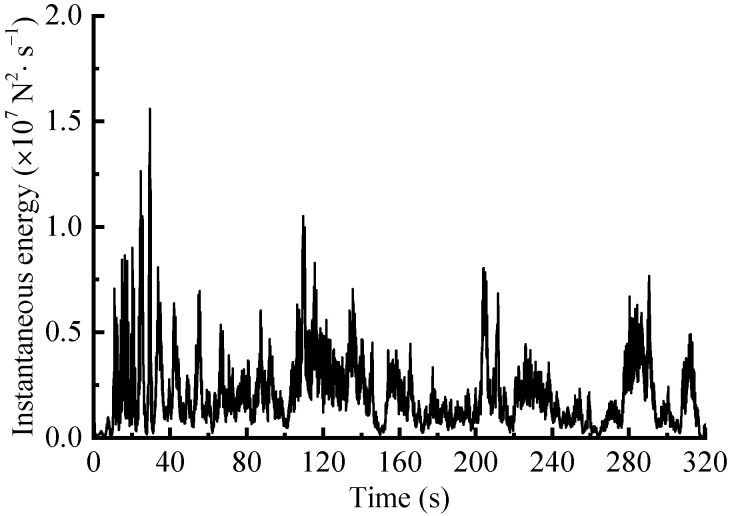
Instantaneous energy spectrum of the spring signal.

As mentioned, to make the points of the instantaneous energy spectrum correspond to the time-domain points of an original discrete-time signal, the ‘time instant’ parameter involved in ‘tfrwv’ function is generally kept as: $T=1:length\left(x_{0}\left(t\right)\right)$. The default setting of the ‘number of frequency bins’ is $2^{n}$ times, which usually requires a combination of calculation accuracy and calculation efficiency. After due consideration, a compromised setting of N=512 is chosen to ensure a reasonable frequency resolution without compromising calculation speed. According to the outlined technical flow, the pre-processed load signal is transformed to obtain the Wigner-Ville spectrum, as depicted in Figure 4. Subsequently, the Instantaneous Energy Spectrum (IES) is computed, as illustrated in Figure 5.

**Figure 6:**
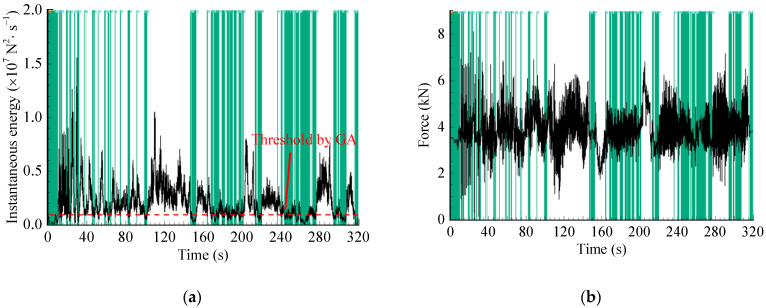
WVT-based editing method: (**a**) Localization of no/minor damage segments in IES; (**b**) Time-domain segments deletion.

**Figure 7:**
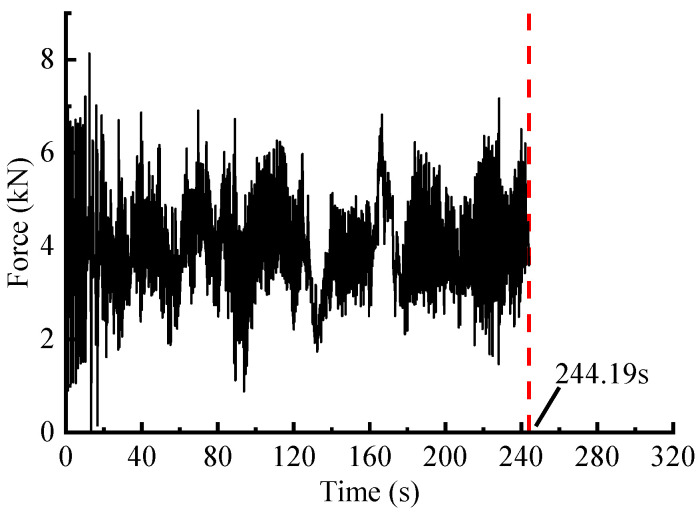
Accelerated spectrum of the force signal via WVT-based editing.

In addition, the optimal threshold $p_{0}$ is calculated using the GA-based method. According to the literature reference [22], some specific parameter settings are as follows: ‘Population size’ is set to 100, ‘Maximum genetic generations’ is set to 100, ‘Crossover type’ is the multi-point crossover, ‘Crossover probabilities’ is set to 0.8, and ‘Mutation probabilities’ are set to 0.1, etc. Then, $p_{0}$ is calculated as $9.0\times 10^{5}\;N^{2}/s$, where the objective function $L\left(p_{0}\right)=0.7631$, and pseudo-damage retention ratio Dam=0.97. After that, the IES is analyzed to locate smaller energy points based on the calculated threshold, as depicted by the shaded area in Figure 6a. Subsequently, these points are mapped to their corresponding time-domain positions in the original signal, as shown in the shaded segments in Figure 6b. Finally, the accelerated spectrum is obtained, as illustrated in Figure 7, with a time duration of 244.19 s.

### 3.3. Accelerated Editing via Time-Domain Editing, STFT-Based and ST-Based Methods

Based on the same pseudo-damage retention (97%) and statistical parameters limitation (<15%), some existing mainstream methods, such as time-domain editing, STFT-based, and ST-based are performed to edit the same signal in order to validate and compare the editing efficiency.

#### 3.3.1. Accelerated Editing via Time-Domain Editing Method

**Figure 8:**
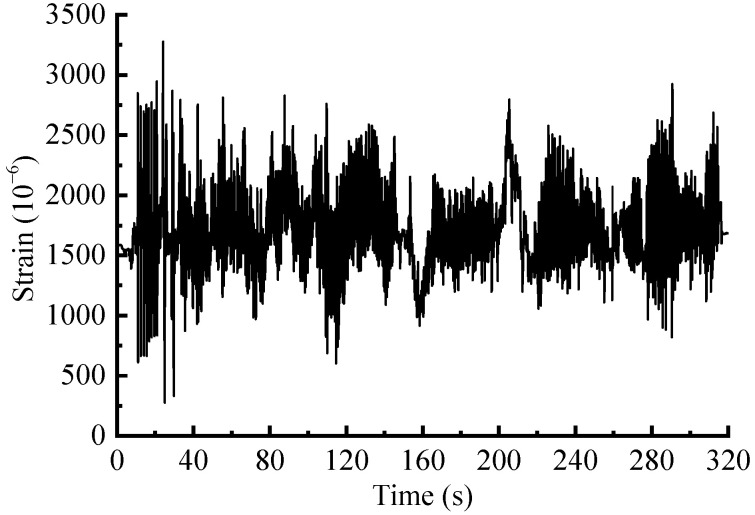
Reference strain signal of the suspension spring.

**Figure 9:**
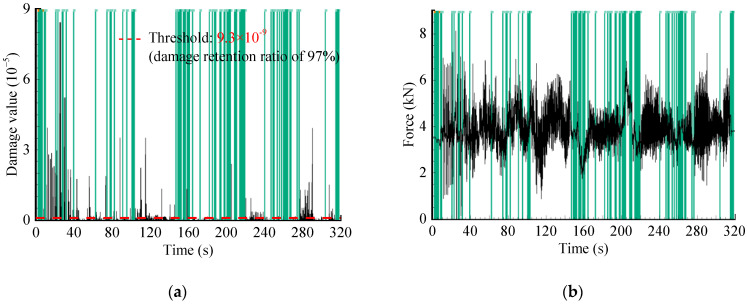
Time-domain editing method: (**a**) Damage–time relationship curve of referenced strain signal; (**b**) Time segments localization of no/minor damage.

**Figure 10:**
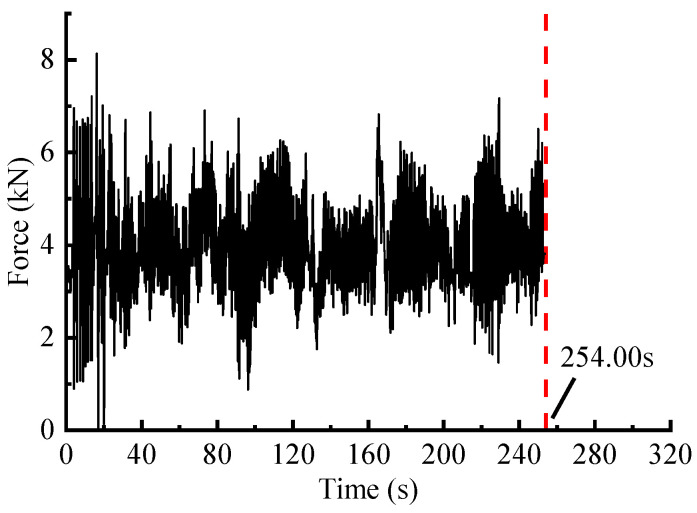
Accelerated spectrum of the force signal.

The time-domain editing process is mainly performed in nCode software. This method generally targets strain or stress signals, as the force spectrum cannot be directly edited [6]. According to Section 3.1, the spring strain signal correlates with the force signal, which means that the strain history range is proportional to the force history range, so the measured strain load was utilized as a reference signal for editing (Figure 8). The signal reduction with specified damage retention could be achieved by setting two key parameters: damage retention ratio (97%) and windows’ length (1.5 s). The time-domain damage distribution is shown in Figure 9a. The threshold was then computed automatically according to the set parameters, and the strain-load time segments with small damage contributions were identified in Figure 9b. The time segments at the same position in the original spectrum were removed. The final obtained accelerated signal is shown in Figure 10, with a time duration of 254 s.

#### 3.3.2. Accelerated Editing via STFT-Based Method

**Figure 11:**
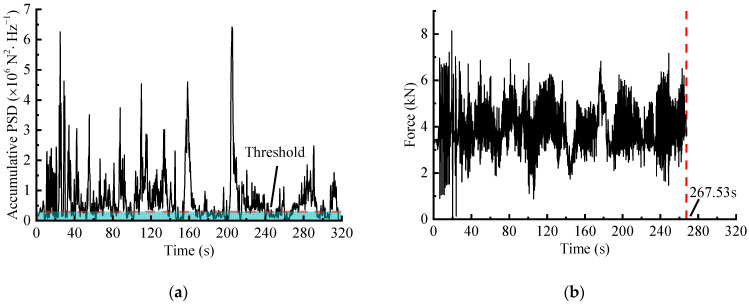
STFT-based editing method: (**a**) AccPSD of load spectrum based on STFT; (**b**) Accelerated spectrum of the force signal.

The STFT-based editing process is with reference to [12]. Combined with a ‘spectrogram’ function in MATLAB, the window function is chosen as the Gaussian, with a window length $N_{fft}=256$ and an overlap $N_{lap}=0.8\times N_{fft}$. Consequently, the AccPSD (Figure 11a) was calculated, and the acceleration threshold was set according to the pseudo-damage retention ratio, so that the segments with high damage contribution were identified and retained. The retained segments with high AccPSD values were then mapped to the original time-domain to extract corresponding time segments, which were spliced as an accelerated spectrum (Figure 11b).

#### 3.3.3. Accelerated Editing via ST-Based Method

**Figure 12:**
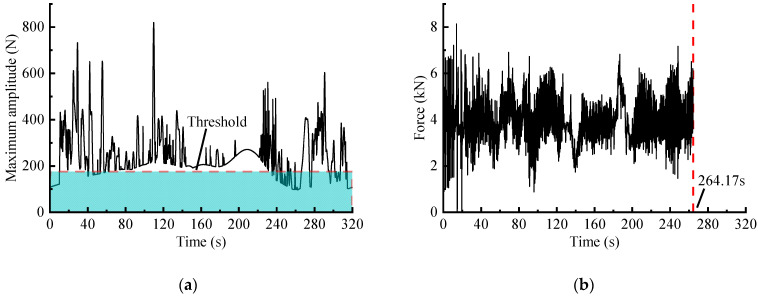
ST-based editing method: (**a**) Maximum amplitude spectrum based on ST; (**b**) Accelerated spectrum of the force signal.

The ST-based editing process is with reference to [24]. The S-transform is performed combined with the ‘st’ function in time-frequency toolbox, with key parameters such as ‘minfreq’ and ‘maxfreq’ determined based on the input signal. The ‘samplingrate’ is commonly set as $c=1/f_{s}$ and the ‘freqsamplingrate d’ represents the frequency-sampling interval in results. The maximum amplitude spectrum (Figure 12a) was extracted from the calculated two-dimensional time-frequency amplitude matrix. After that, an acceleration threshold with the same pseudo-damage retention ratio was set to obtain the accelerated spectrum (Figure 12b).

## 4. Results and Comparative Analysis

In the engineering application, the load characteristics are mainly evaluated in terms of time duration, damage value, statistical characteristic parameter, power spectral density (PSD), and level crossing count distribution [36]. Since the quality of the accelerated signals greatly affects the reliability of the fatigue analysis results, the following section provides a detailed comparison between the accelerated and original signals across the aforementioned aspects.

### 4.1. Time Duration and Damage Retention

Time durations and amount of pseudo-damage retention based on accelerated spectrums obtained by various mentioned methods are listed in Table 1.

In the case that the damage retentions is about 97%, the time duration of the accelerated signals obtained through WVT-based, time-domain editing, STFT-based and ST-based methods are 244.19 s, 254.00 s, 267.53 s and 264.17 s, respectively. These values correspond to time duration ratios of approximately 76.30%, 79.38%, 83.50%, and 82.55% compared to the original signal. The results shows that the WVT-based editing method exhibits a significant time-length compression effect similar to the other three methods.

### 4.2. Statistical Parameters and Errors

The errors of several statistical parameters of load spectrums are adopted to measure the effectiveness of editing. Table 2 summarizes the statistical measures of the original and edited force signals from the proposed and the other three methods, as well as the corresponding percentage errors.

The results demonstrate that, compared to the original, a large number of small-amplitude stress cycles are removed from the accelerated signal around the mean value, causing the value to increase. Besides, the mean value of that from WVT-based editing is the largest because many of the smallest amplitudes are eliminated. Simultaneously, the kurtosis decreases because the occurrence probability of small values around the mean diminishes, i.e., the probability density distribution curve had a reduced peak around the mean. In addition, the deletion of small amplitude cycles results in a larger load energy per unit of time, resulting in an increase in RMS. Notably, the WVT-based accelerated signal exhibits the largest compression ratio, resulting in some relatively greater changes in its statistical parameters. Nonetheless, the error values remain below 15%, which shows good agreement with the original in the statistical distribution.

### 4.3. Power Spectral Density

PSD is commonly utilized to assess the distribution of signal power across different frequencies in the frequency-domain analysis. The PSD of loads reflects the effect of energy carried by loads at different frequencies on component damage. Since the edited signal is shorter than the original, the load energy tends to increase [22]. In addition, for PSDs of two random loads, the Pearson correlation coefficient [1] can be used to analyze the consistency of the changing trend, which is calculated as:

(13) $$r=\frac{M\sum_{i=1}^{M}x_{i}y_{i}-\sum_{i=1}^{M}x_{i}\sum_{i=1}^{M}y_{i}}{\sqrt{M\sum_{i=1}^{M}x_{i}^{2}-{\left(\sum_{i=1}^{M}x_{i}\right)}^{2}}\sqrt{M\sum_{i=1}^{M}y_{i}^{2}-{\left(\sum_{i=1}^{M}y_{i}\right)}^{2}}}$$

where $x_{i},\;i=1,2,\cdots M$ (M is the amount of data) denotes the PSD of the original load, and $y_{i},\;i=1,2,\cdots M$ denotes that of the accelerated load. The closer the value of r is to 1, the better the fit between the two curves. Two load signals are generally considered to possess a strong positive phase relationship when |r| is greater than 0.8 [37].

**Figure 13:**
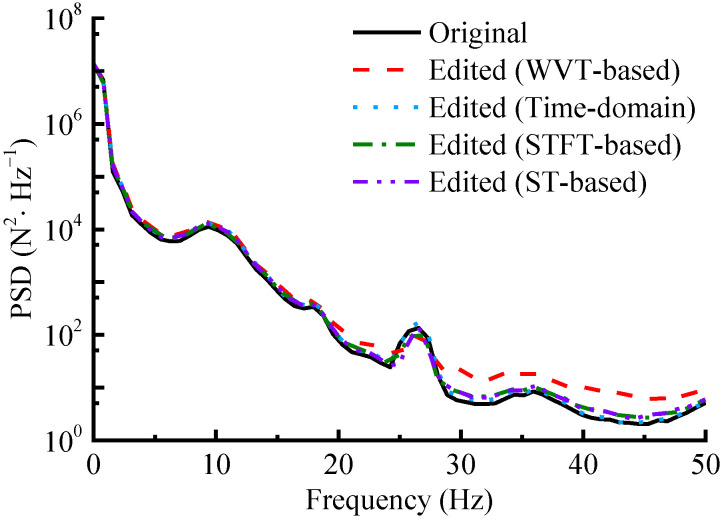
The PSD distribution curves.

As shown in Figure 13, the PSD distributions and change trends of the accelerated spectrums in the low-frequency part are almost the same as the original, while the removal of small-amplitude loads in the high-frequency band causes the PSD distributions in this region to vary. Notably, the WVT-based edited spectrum, which has a greater compression in time duration, shows a more pronounced upward shift in its PSD distribution curve in the high-frequency band. Nevertheless, this shift is considered acceptable. Meanwhile, the Pearson correlation coefficients between the PSD of the original and that of the accelerated spectrums obtained via WVT-based, time-domain editing, STFT-based and ST-based methods are calculated as 0.990, 0.999, 0.995 and 0.996, respectively. These coefficients exceed 0.99 and approach 1, indicating a very high degree of agreement. These findings demonstrate that the proposed accelerated editing effectively avoids changing frequency-domain characteristics, validating the effectiveness of the WVT-based method.

### 4.4. Level Crossing Analysis

**Figure 14:**
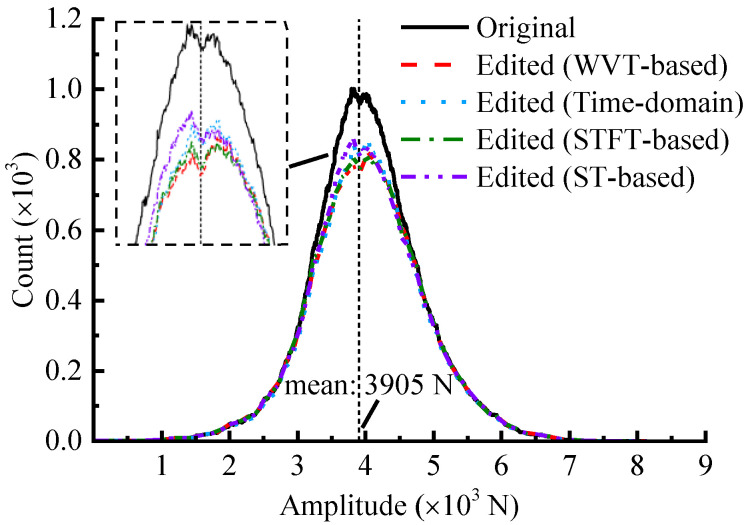
The level crossing counts.

For amplitude-domain analysis, the number of level crossings, defined as the number of times the load magnitude crosses a specific value, is also used to evaluate the editing quality [1]. The signal distribution at each load level is reflected by counting the number of level crossings as the sample point rises or falls, which can evaluate the difference in amplitude distributions of different load spectrums. Figure 14 illustrates the level counts of several load signals.

The results indicate that, compared to the original spectrum, the counts of the edited spectrums are significantly reduced for small amplitude cycles, while they are essentially the same as the original for large amplitude cycles. In other words, the no-damage or low-damage contribution loads are greatly reduced, while the high-damage contribution loads are well preserved, ensuring the damage effectiveness after editing. Furthermore, for the counts of small-amplitude cycle counts around the mean value, the WVT-based accelerated spectrum exhibits a greater reduction compared to the other methods, showing a greater acceleration efficiency.

From the results presented above, it can be deduced that the proposed editing method based on the Wigner-Ville transform can maintain nearly the same damage potential as that of the original spectrum while reducing the time durations for efficient and accurate durability test analysis.

## 5. Fatigue Life Simulation Analysis

To further validate the proposed method for fatigue life test and damage distributions, the fatigue life and damage distribution of the spring signal were estimated using nCode software.

**Figure 15:**
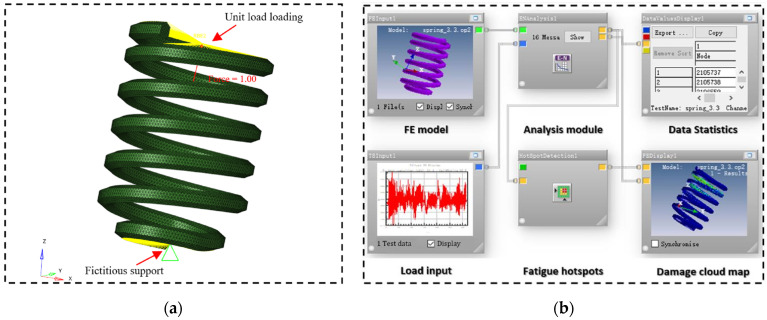
Fatigue simulation analysis: (**a**) Finite element model; (**b**) The analysis flow diagram.

The reliable finite element model of the suspension coil spring was built in HyperMesh software (HW 2019), with the element type being first-order tetrahedral mesh and the element size about 3 mm. After the model was built, a fictitious support was defined to constrain at the bottom, while a unit axial force was applied to the top of the model. The stress distribution of the unit load was calculated via the OptiStruct solver employing the inertial release analysis method. Subsequently, after importing the static analysis results into nCode, the damage and fatigue life of the suspension coil spring under random load were calculated by replacing the unit load with the spectrums (original and edited) as load history input. Figure 15 illustrates the finite element model and fatigue simulation method. The spring’s material is high-strength spring steel. For fatigue simulation, a material of SAE 1045 steel from the nCode material library was selected, with ultimate tensile strength 751 MPa, yield strength 516 MPa, and modulus of elasticity 2.07 × 105 MPa.

The statistics of the fatigue simulation results are listed in Table 3, using several signals as input in turn. The fatigue life distributions and failure positions based on each accelerated signal are almost consistent with the original because the pseudo-damage retention ratio is as high as 97%. The number of life cycles of the accelerated spectrum via WVT-based editing is almost the same as that of the original, indicating that the proposed method avoids erroneously deleting time segments with significant damage contribution while simultaneously preserving the original loading characteristics. Furthermore, the simulation calculation time is directly proportional to the amount of the loading data. As the WVT-based accelerated spectrum undergoes the highest time duration compression, its simulation calculation time can be significantly reduced. In conclusion, the WVT-based editing method can more effectively improve the analysis efficiency based on ensuring the accuracy of fatigue analysis.

## 6. Conclusions

This paper proposed a WVT-based accelerated editing method for the load-time history of vehicle components. An IES information of the original signal is obtained via the Wigner-Ville time-frequency transform. The threshold is determined using the GA to identify and eliminate ineffective damage contributing time-domain points to obtain an accelerated signal. The proposed and some existing methods are applied to edit the same example of a spring signal to compare the effects. The main conclusions are summarized as:

(1) The time duration of the accelerated loads obtained by the proposed method is shorter than that of others. Furthermore, the errors of the statistical parameters between the edited and original spectrums of numerous editing methods are all below 15%, and both the PSD distributions and level crossing counts align well with the original. These results indicate that the WVT-based editing method can avoid changes in frequency-domain characteristics while removing more small-amplitude loads.
(2) Fatigue simulation results reveal that edited load signals provide the same loading effect as the original. Furthermore, the WVT-based editing can compress the time duration as much as possible, based on ensuring that the damage characteristics are consistent with the original.
(3) The proposed method effectively avoids the deviations in the analysis results of random loads caused by the inherent defects of the STFT-based method with fixed window width, the WT-based method with difficult-to-determine decomposition functions and layers, and the unstable solution of the ST-based method. It achieves a more efficient accelerated editing quality, which provides a reasonable and efficient reference basis for the load compilation in vehicle durability tests. Similarly, the proposed method can be extended to the process of other signals, such as wheel center sextant force and strain, etc.

## Figures and Tables

**Table 1:** Time durations and pseudo damage retention ratios of the edited and original load spectrums.

Methods	Time Duration/s	Time Duration Ratio/%	Pseudo-Damage Retention Ratio/%
Original	320	--	--
WVT-based	244.19	76.30	97.00%
Time-domain	254.00	79.38	97.00%
STFT-based	267.53	83.50	97.10%
ST-based	264.17	82.55	96.97%

**Table 2:** Statistical parameters and relative errors of the results of several editing methods.

Methods and Statistical Parameters’ Errors	Mean/*N*	Error/%	Kurtosis	Error/%	RMS Value	Error/%
Original	3905	--	4.20	--	3977	--
WVT-based	3960	1.4	3.62	13.8	4045	1.7
Time-domain	3948	1.1	4.00	4.8	4024	1.2
STFT-based	3923	0.5	3.70	12.0	4005	0.7
ST-based	3933	0.7	3.86	8.1	4013	0.9

**Table 3:** Fatigue simulation analysis results.

Loading	Fatigue Damage Value	Fatigue Life (Times)	Fatigue Failure Point (No.)	Simulation Duration (s)
Original	7.7850 × 10^−5^	1.2845 × 10^4^	2,105,737	137.15
WVT-based	7.7850 × 10^−5^	1.2845 × 10^4^	2,105,737	95.03
Time-domain	7.7820 × 10^−5^	1.2852 × 10^4^	2,105,737	100.96
STFT-based	7.7840 × 10^−5^	1.2847 × 10^4^	2,105,737	106.46
ST-based	7.7820 × 10^−5^	1.2852 × 10^4^	2,105,737	105.41

## Data Availability

The data used to support the findings of this study are available from the corresponding author upon request.
